# Ti_3_C_2_T_x_ as a Sensor for SF_6_/N_2_ Nitrogen-Containing Fault Decomposition Characteristic Products: A Theoretical Study

**DOI:** 10.3390/nano12132311

**Published:** 2022-07-05

**Authors:** Fuping Zeng, Hao Qiu, Xiaoxuan Feng, Xianzong Chao, Liangjun Dai, Qiang Yao, Ju Tang

**Affiliations:** 1 School of Electrical Engineering and Automation, Wuhan University, Wuhan 430072, China; elvis_qiu@icloud.com (H.Q.); xiaoxuanf@whu.edu.cn (X.F.); 2018302070035@whu.edu.cn (X.C.); cqtangju@vip.sina.com (J.T.); 2 Hubei Key Laboratory of Power Equipment & System Security for Integrated Energy Resources, Wuhan 430072, China; 3 Electric Power Research Institute, State Grid Chongqing Electric Power Company, Chongqing 401123, China; 2018302070058@whu.edu.cn (L.D.); yaoqiang212@aliyun.com (Q.Y.); 4 State Key Laboratory of Power Transmission Equipment & System Security and New Technology, Chongqing University, Chongqing 400044, China

**Keywords:** Ti_3_C_2_T_x_, SF_6_/N_2_ decomposition characteristic products, gas sensor, DFT

## Abstract

The SF_6_/N_2_ gas mixture is an alternative gas to SF_6_. SF_6_/N_2_ will decompose and generate nitrogenous characteristic gases, such as NO, NO_2_, N_2_O, and NF_3_, when exposed to long-term partial discharge. The adsorption models of Ti_3_C_2_T_x_ (T=O, F, OH) and NO, NO_2_, N_2_O, NF_3_ were constructed, and the most stable adsorption structure was selected in this paper. The electron density and density of states of the adsorption system were further analyzed to study the adsorption behavior, and the sensing performance was evaluated in the end. The results are as follows: four gases could be spontaneously adsorbed on Ti_3_C_2_T_x_, and strong adsorption occurred when surface terminal groups were OH, forming hydrogen or chemical bonds with significant charge transfer. Results show that Ti_3_C_2_(OH)_2_ had a stronger sensing ability than Ti_3_C_2_F_2_ and Ti_3_C_2_O_2_. The conductivity of the Ti_3_C_2_T_x_ with different terminal groups was improved after the adsorption of NO and NO_2_, showing Ti_3_C_2_T_x_ had a good sensing ability for NO and NO_2_. It was difficult for the four gases to desorb from the Ti_3_C_2_(OH)_2_ surface, but the adsorption on the Ti_3_C_2_F_2_, Ti_3_C_2_O_2_ surface had a short recovery time at room temperature.

## 1. Introduction

SF_6_ has been widely used in high-voltage electrical equipment because of its excellent insulation and arc extinguishing properties. However, SF_6_ is a greenhouse gas, and its global warming potential is 23,900 times that of CO_2_, and it can exist in the atmosphere for more than 3200 years. It is one of the six greenhouse gases prohibited by the Kyoto Protocol [1,2,3]. In order to relieve the environmental pressure brought about by SF_6_, the power industry has used the SF_6_/N_2_ gas mixture as an insulation medium. SF_6_/N_2_ not only has good insulation performance but also solves the problem of high SF_6_ liquefaction temperature. It can effectively reduce the use and emission of SF_6_ and relieve the greenhouse effect of SF_6_ [4,5,6].

In the long-term running of power equipment, various insulation defects will unavoidably occur, and insulation faults, such as partial discharge or overheating, will be generated under the combined action of voltage and current. SF_6_/N_2_ will gradually decompose under the continuous action of these faults, generating not only sulfur-containing characteristic gases, such as SO_2_, SOF_2_, SO_2_F_2_, and H_2_S [7], but also nitrogen-containing characteristic gases, such as NO, NO_2_, N_2_O, and NF_3_ [8,9,10]. Since the SF_6_/N_2_ fault decomposition process is directly related to the fault properties, the internal insulation fault of the equipment can be detected in time by monitoring fault decomposition characteristic products, which is essential for the safe operation of gas-insulated equipment [11,12].

Two-dimensional layered nanomaterials have excellent electrical, mechanical, and optical properties due to their unique structure, and their high specific surface area is conducive to gas adsorption and sensing [13,14,15,16,17]. Two-dimensional transition metal carbides, carbon and nitrogen compounds (collectively called MXene) have received a lot of attention and research from researchers since their discovery in 2011. MXene has the chemical formula M_n+1_X_n_T_x_ (*n* = 1–3), where M represents the transition metal (such as Sc, Ti, Zr, Hf, V, Nb, Ta, Cr, Mo, etc.), X is carbon or nitrogen, and T_x_ stands for terminal group, such as fluorine, oxygen, or hydroxyl. Ti_3_C_2_T_x_ was the first MXene synthesized and has been used in many sensing-related studies.

Eunji Lee et al. [18] prepared a sensor of Ti_3_C_2_T_x_ and successfully detected all the tested volatile organic compound gases at room temperature. Then, they proposed a possible sensing mechanism for the sensor by analyzing the interaction between the gas and the majority carrier of the material. Chen et al. [19] chose Ti_3_C_2_T_x_ and WSe_2_ as materials for hybridization and prepared the Ti_3_C_2_T_x_/WSe_2_ hybrid sensor with low noise level and ultra-fast response and recovery time. It has high sensitivity and selectivity for detecting oxygenated volatile organic compounds. Wu et al. [20] realized the detection of NH_3_ by Ti_3_C_2_T_x_ at room temperature and investigated its highly selective adsorption behavior using the density functional theory (DFT) calculations. Kong et al. [21] adsorbed SF_6_ decomposition gas on Ti_3_C_2_T_x_ using Quantum Espresso software and modified the surface of Ti_3_C_2_T_x_ by adding atomic vacancies. The results showed that Ti_3_C_2_T_x_ with dot vacancies can detect SF_6_ decomposition products with high sensitivity and low electronic noise, and the sensitive detection ability for SO_2_ is especially obvious.

Based on the previous research on gas-sensitive sensing of Ti_3_C_2_T_x_ for sulfur-containing gases [22], this paper performed first-principle calculations on the adsorption behavior of SF_6_/N_2_ nitrogen-containing fault decomposition characteristic components on the surface of Ti_3_C_2_T_x_ with different terminal groups, and adsorption morphology, adsorption distance, adsorption energy, charge transfer, electron density, and density of states were analyzed to explore the gas sensing ability of Ti_3_C_2_T_x_ with different terminal groups for NO, NO_2_, N_2_O, and NF_3_.

## 2. Computational Methods

This paper is mainly based on the DFT. The simulation calculations regarding the adsorption system of gas molecules with Ti_3_C_2_T_x_ are carried out in the DMol^3^ module of Material Studio [23]. In this paper, a 3 × 3 × 1 periodic supercell of Ti_3_C_2_T_x_ is established. Firstly, NO, NO_2_, N_2_O, and NF_3_ gases are placed on the surface of Ti_3_C_2_T_x_, and multiple directions and different possible sites of gas molecules adsorption are considered. Then, the most stable adsorption site of gas molecules is finally selected for further analysis according to the energy of the system. The K-point settings of the Brillouin zone for structure optimization and electronic property calculation are 4 × 4 × 1 and 8 × 8 × 1, respectively, and the optimization of the structure, energy, and related properties of the gas–solid interface system are calculated by the GGA-PBE method [24,25]. The DNP basis group is chosen for the expansion of the electronic wave function [26], and the Grimme method in the DFT-D dispersion correction is used to describe the van der Waals interaction forces [27,28]. For the paramagnetic molecules NO, NO_2_, and N_2_O, the computational setup takes into account the spin polarization [29]. The all-electron model is used for the core treatment of the gas molecules, and the DFT Semi-core pseudopotential (DSPP for short) is used for the solid surface. The energy convergence threshold, the maximum force threshold, and the maximum displacement threshold for geometric optimization are set to 2.0 × 10^−5^ Ha, 0.004 Ha/Å, and 0.005 Å, respectively, and the convergence accuracy of SCF is 1.0 × 10^−5^. The direct inversion DIIS value in the SCF iterative subspace is set to 6, and the smearing value of the thermal tailing effect is set to 0.005 Ha. In addition, to eliminate the interaction of adjacent layers, a 25 Å vacuum layer is set in the z-direction. The strength of the interaction between the gas molecules and the sensing material is expressed by the adsorption energy (*E_ads_*) as follows:

*E*_*ads*_ = *E*_*total*_ − *E*_*gas*_ − *E*_*substrate*_

where $E_{total}$, $E_{gas}$, and $E_{substrate}$ represent the total energy of the gas/Ti_3_C_2_T_x_ adsorption system, isolated gas molecule, and pristine Ti_3_C_2_T_x_, respectively.

*Q_t_* represents the charge transfer between the gas molecule and Ti_3_C_2_T_x_ during the adsorption as follows:

*Q*_*t*_ = *Q*_1_ − *Q*_2_

where *Q*_1_ and *Q*_2_ represent the total charges of the adsorbed and the isolated gas molecules. A negative value of *Q_t_* means the electrons’ transfer from the substrate to gas molecules.

## 3. Results and Discussion

### 3.1. Gas Adsorption on Ti_3_C_2_F_2_

**Figure 1:**
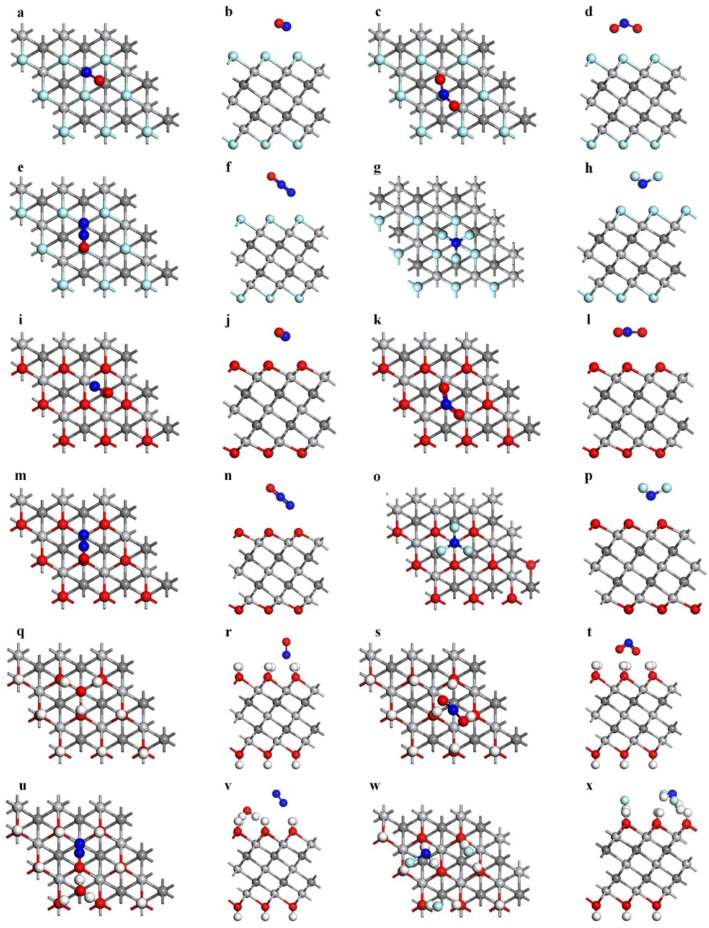
The most stable adsorption structures of NO, NO_2_, N_2_O, and NF_3_ on (**a**–**h**) Ti_3_C_2_F_2_, (**i**–**p**) Ti_3_C_2_O_2_, and (**q**–**x**) Ti_3_C_2_(OH)_2._ (Light gray–Ti, dark grey–C, cyan–F, red–O, blue–N, white–H).

When gas molecules make contact with Ti_3_C_2_F_2_ surface, adsorption may occur at different sites. The most stable adsorption sites of gas molecules and the orientation of adsorption on Ti_3_C_2_F_2_ were selected according to the value of adsorption energy, as shown in Figure 1, where Figure 1a–h show the top and side views of the gas after adsorption. The structure after adsorption shows that no significant changes occur in the gases as well as the substrate material during the adsorption process. The adsorption distances of gas molecules with Ti_3_C_2_F_2_ are all between 2.8 Å and 3.0 Å, as shown in Table 1. After measurement and comparison, the bond length of the adsorbed gas molecules does not change significantly, and the specific values of the bond length change are shown in Table 1 (The original bond lengths of the four gas molecules are shown in Figure S1). Table 2 gives the adsorption energies of the four gas molecules on the best adsorption sites of Ti_3_C_2_F_2_, and the adsorption energies of NO, NO_2_, N_2_O, and NF_3_ are −0.216 eV, −0.213 eV, −0.273 eV, and −0.323 eV, respectively. The negative adsorption energies indicate that the adsorption of gases does not require external energy.

**Figure 2:**
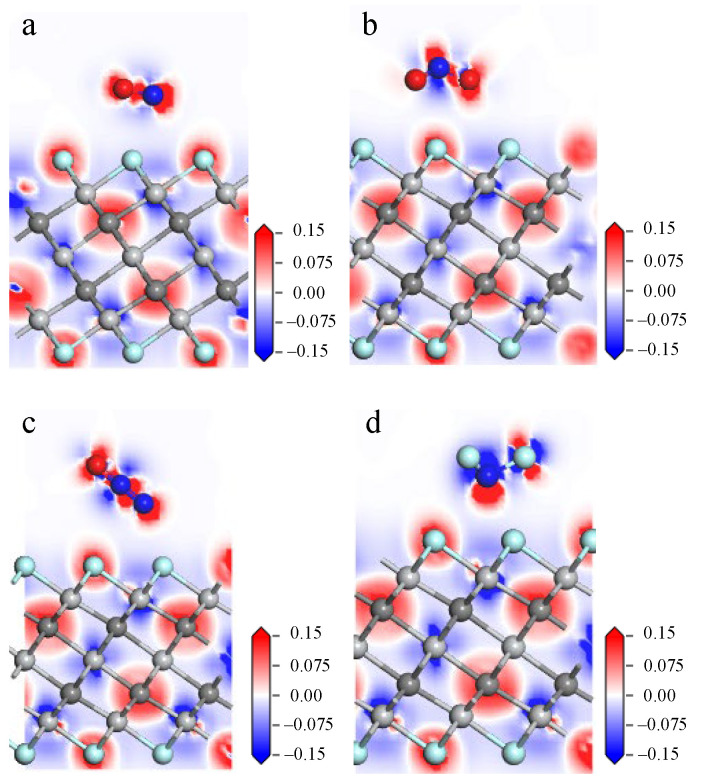
Deformation charge density diagrams of (**a**) NO, (**b**) NO_2_, (**c**) N_2_O, (**d**) NF_3_ on Ti_3_C_2_F_2_ surface.

Electron density represents the probability of finding electrons at specific locations around atoms or molecules. The deformation charge density is the difference between the electron density after bonding and the atomic charge density at the corresponding point. By calculating and analyzing the deformation charge density, one can understand the bonding of the atoms in the system and the charge transfer during the bonding process. Figure 2 lists the deformation charge density diagrams for the NO, NO_2_, N_2_O, and NF_3_ adsorption models (red represents electron gain, and blue represents electron loss). There is not much charge aggregation between the gas molecules and the atoms on the material surface, as shown in the four pictures below, indicating a weak bonding behavior between them. In addition, the charge transfer values in the adsorption structure are given in Table 2. NO, N_2_O, and NF_3_ lose 0.111 e, 0.008 e, and 0.014 e, respectively, while NO_2_ gains 0.129 e from the surface of Ti_3_C_2_F_2_, and NO and NO_2_ have a large charge transfer in the process of adsorption.

**Figure 3:**
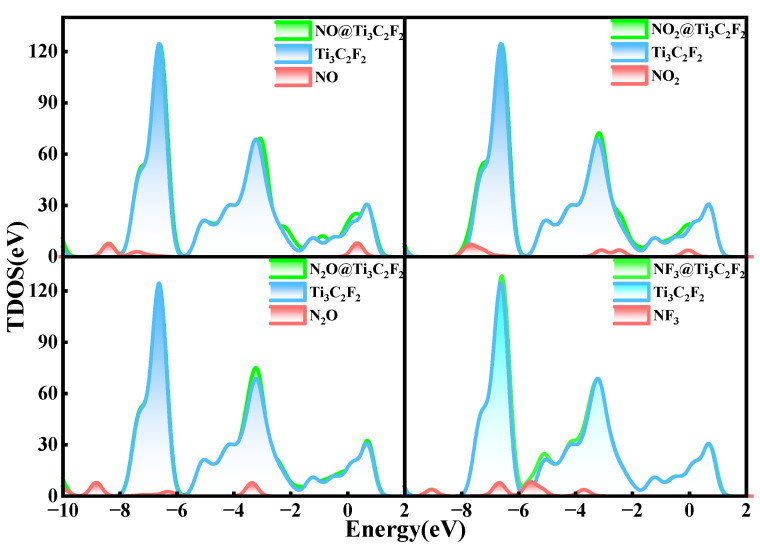
Total electron density of states of Ti_3_C_2_F_2_ adsorbing NO, NO_2_, N_2_O, and NF_3_.

To further clarify the electronic structure characteristics of the adsorption systems, the total density of states (TDOS) and partial density of states (PDOS) of all adsorption systems are analyzed. The TDOS plots of Ti_3_C_2_F_2_ after gas adsorption are shown in Figure 3. The density of states of the whole system after the adsorption of NO and NO_2_ experiences a significant increase near the Fermi energy level, indicating that the adsorption of these two gases enhances the conductivity of the material. This phenomenon does not occur after the adsorption of N_2_O and NF_3_. As seen from the figure, the effects of N_2_O and NF_3_ on the whole adsorption system are below −3 eV, and the density of states of the whole system does not change greatly after adsorption. So, the adsorption of these two gases does not significantly enhance the conductivity of the material. These findings are consistent with the value of the charge transfer reflected in Table 2.

**Figure 4:**
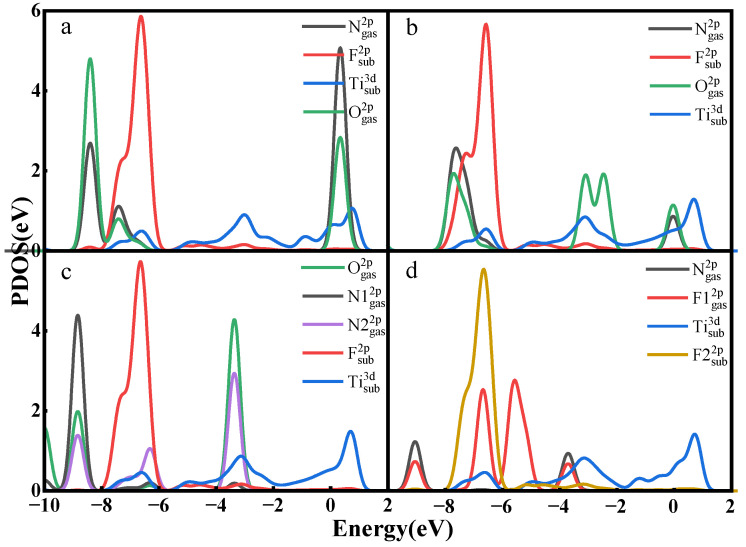
Partial electron density of states of Ti_3_C_2_F_2_ adsorbing (**a**) NO, (**b**) NO_2_, (**c**) N_2_O, (**d**) NF_3_.

In the PDOS of Figure 4, only a small part of the orbital overlaps between the atoms of NO, NO_2_, and N_2_O and the atoms of the material surface, indicating that the interactions between the atoms are not strong. The F 2p orbital of NF_3_ has a partial overlap with the F 2p orbital of the surface around −7 eV, indicating that there are some interactions between them but not the formation of a strong chemical bond. Taking all the analyses together, the adsorption of the gases on the Ti_3_C_2_F_2_ surface is a physical adsorption, and there is no new chemical bond formed between the atoms composing the gas molecules and the atoms on the material surface.

### 3.2. Gas Adsorption on Ti_3_C_2_O_2_

Figure 1i–p are top and side views of the four gas molecules after adsorption on the Ti_3_C_2_O_2_ surface. The pictures show that there is no significant structural change in the gas and the substrate material after adsorption occurs, and the bond length changes of the gas molecules are measured, as shown in Table 1, which further confirms that there is only a slight change in the molecular structure after adsorption. Table 1 also shows that the gas adsorption distances are all within the range of 2.7 Å–3.1 Å. From the slight structural changes and large adsorption distances, it can be assumed that the adsorption of gases on Ti_3_C_2_O_2_ is a physical adsorption. The adsorption energies of the four gases on the Ti_3_C_2_O_2_ surface are given in Table 2 (−0.507 eV, −0.115 eV, −0.240 eV, and −0.386 eV for NO, NO_2_, N_2_O, and NF_3_, respectively). All have negative adsorption energies, as in the case of adsorption on the Ti_3_C_2_F_2_ surface, indicating that the adsorption of the gases does not require energy from outside.

**Figure 5:**
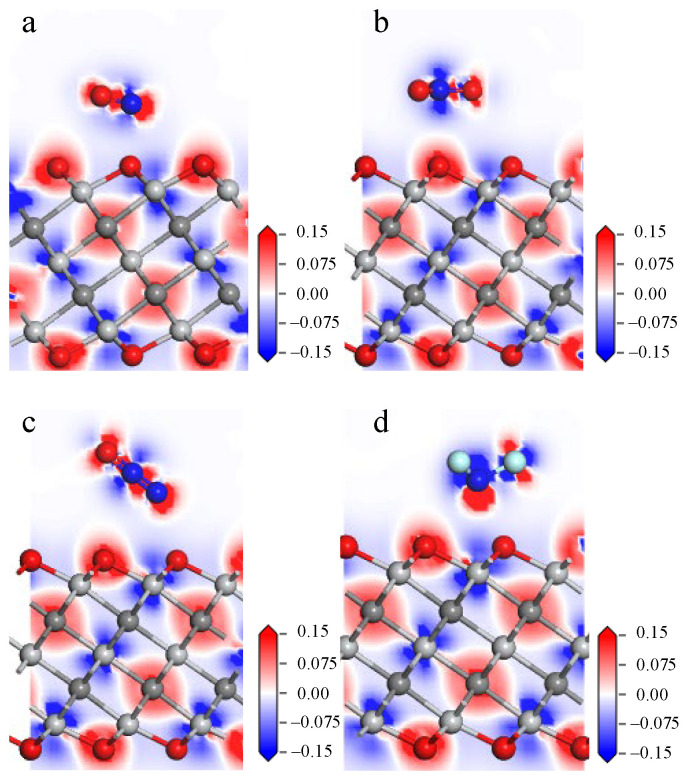
Deformation charge density diagram of (**a**) NO, (**b**) NO_2_, (**c**) N_2_O, (**d**) NF_3_ on Ti_3_C_2_O_2_ surface.

The deformation charge density plots of the four adsorption systems are shown in Figure 5, which shows that the aggregation of electrons between the atoms of the four gases and the material surface is difficult due to the long adsorption distance, indicating that they have difficulty in forming chemical bonds with strong interactions. The charge transfer given in Table 2 shows that 0.284e is transferred from NO to Ti_3_C_2_O_2_ surface, 0.077 e from NO_2_ to Ti_3_C_2_O_2_ surface, 0.008 e from Ti_3_C_2_O_2_ surface to N_2_O, and 0.036 e from NF_3_ to Ti_3_C_2_O_2_ surface. Compared with adsorption on Ti_3_C_2_F_2_ surface, the changes of NO and NO_2_ charge transfer are more obvious, with enhanced adsorption of NO and more charge transfer, and weakened adsorption of NO_2_ and less charge transfer.

**Figure 6:**
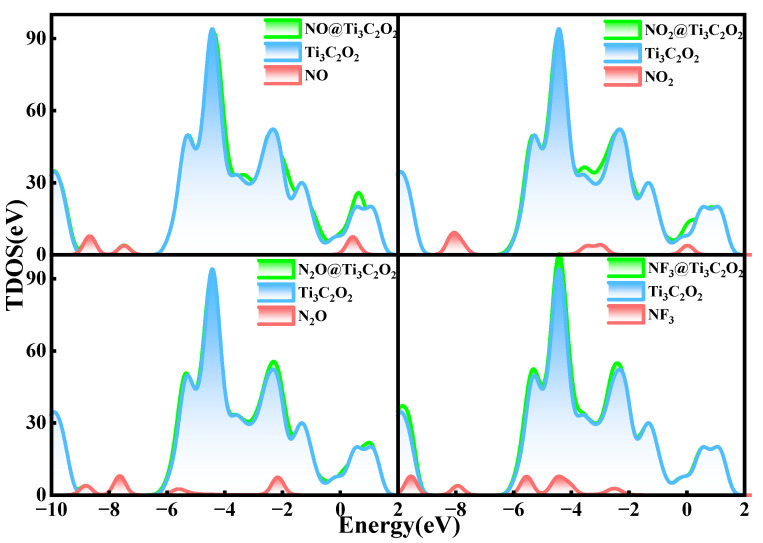
Total electron density of states of Ti_3_C_2_O_2_ adsorbing NO, NO_2_, N_2_O, and NF_3_.

**Figure 7:**
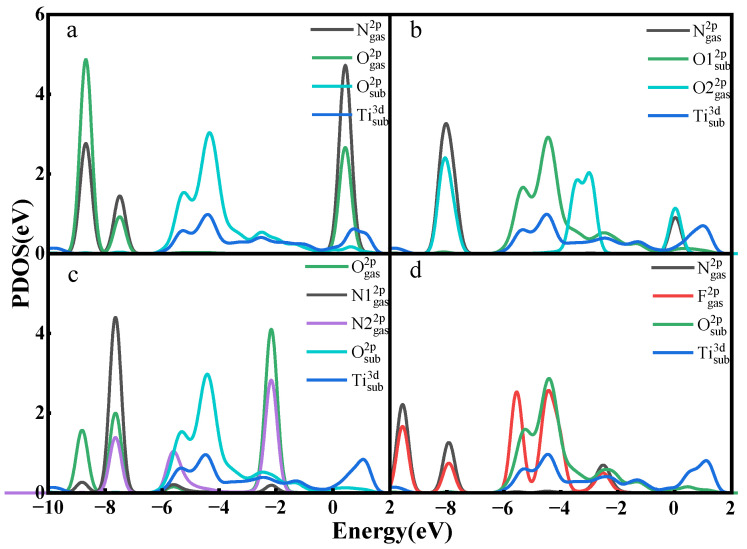
Partial electron density of states of Ti_3_C_2_O_2_ adsorbing (**a**) NO, (**b**) NO_2_, (**c**) N_2_O, (**d**) NF_3_.

Figure 6 shows the TDOS of the whole adsorption system of Ti_3_C_2_O_2_ after gas adsorption. Similar to Ti_3_C_2_F_2_, after the adsorption of NO and NO_2_, the density of states of the system experiences a significant increase near the Fermi energy level, which indicates that the adsorption of these two gases enhances the electrical conductivity of the material. Meanwhile, the adsorption of N_2_O and NF_3_ only makes the density of electronic states at some lower energy levels increase a little, and the effect on the density of states of the system is not great, which also reflects that NO and NO_2_ have a larger charge transfer, and N_2_O and NF_3_ have a lower charge transfer. In the PDOS of Figure 7, only a small part of the orbital overlap between the atoms of NO, NO_2_, and N_2_O and the atoms of the material surface occurs at some energy levels, indicating a weak interaction between the atoms, while the F 2p orbital of NF_3_ has a partial overlap with the O 2p orbital of the surface at around −4.5 eV, indicating some interaction between them. In general, the adsorption of all the four gases on the Ti_3_C_2_O_2_ surface is a physical adsorption.

### 3.3. Gas Adsorption on Ti_3_C_2_(OH)_2_

The top and side views of the gases after adsorption on the best adsorption site on Ti_3_C_2_(OH)_2_ are shown in Figure 1q–x. Unlike the adsorption on Ti_3_C_2_F_2_ and Ti_3_C_2_O_2_, it can be seen that after the adsorption of NO and NO_2_, some H atoms around the gas molecules significantly deflect due to the attraction of the gas molecules. Other H atoms of the surface have different degrees of movement. The N atom of NO is attracted to the H on the surface in Figure 1r, and the O of NO_2_ is attracted to the H on the surface in Figure 1t, and the bond angle has an obvious change. So, the adsorption distances of these two gases with the Ti_3_C_2_(OH)_2_ surface shown in Table 1 are significantly smaller than those of Ti_3_C_2_F_2_ and Ti_3_C_2_O_2_. In addition, the bond length changes of the gases in Table 1 are also greater due to the stronger interactions between the gas and the surface. This strong adsorption is even more significant after the adsorption of N_2_O and NF_3_. Figure 1u,w show that the H atoms on the Ti_3_C_2_(OH)_2_ surface are stripped from the surface and form new chemical bonds with the atoms of gases. The N_2_O reacts with the H atoms of the surface to form H_2_O and N_2_. After NF_3_ adsorbs on the surface, two HF molecules form. All these figures indicate a stronger chemisorption occurring among them. The adsorption energies in Table 2 also reflect the phenomena above. It shows NF_3_ having the strongest interaction with the surface, with an adsorption energy of −9.065 eV, followed by N_2_O with an adsorption energy of −5.461 eV, and finally NO_2_ and NO with adsorption energies of −3.806 eV and −1.709 eV, respectively. Compared to the previous two materials with different terminal groups, the adsorption between four gases and Ti_3_C_2_(OH)_2_ is enhanced in different degrees. 

**Figure 8:**
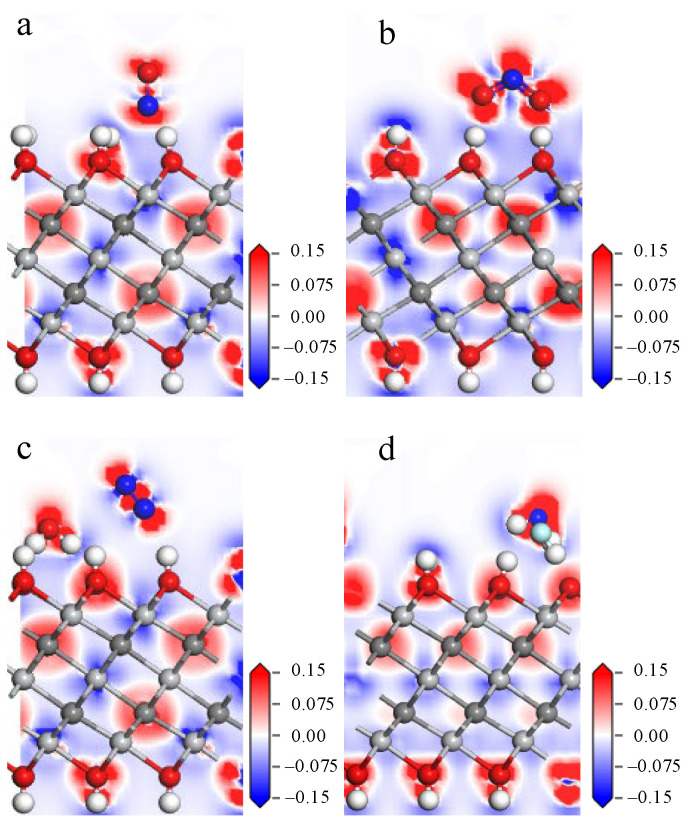
Deformation charge density diagram of (**a**) NO, (**b**) NO_2_, (**c**) N_2_O, (**d**) NF_3_ on Ti_3_C_2_(OH)_2_ surface.

The interaction of the gas molecules with the Ti_3_C_2_(OH)_2_ surface is further analyzed by deformation charge density, and the deformation charge density diagram of the adsorption system is shown in Figure 8. The N atom of NO in Figure 8a is closer to the H atom on the surface, and the red charge aggregation shown between them indicates a stronger interaction. Combined with Table 2, 0.607 e is transferred from the Ti_3_C_2_(OH)_2_ surface to NO. There is also more charge aggregation between the O atom of NO_2_ and the H atom on the surface in Figure 8b, with 0.753 e transferred from the Ti_3_C_2_(OH)_2_ surface to NO_2_. In Figure 8c,d, it can be seen that a large amount of charge aggregates between the H atoms, which are removed from the surface, and the atoms of the gas, further demonstrating the formation of chemical bonds between them, accompanied by 0.707 e and 1.428 e transferring from Ti_3_C_2_(OH)_2_ to N_2_O and NF_3_.

**Figure 9:**
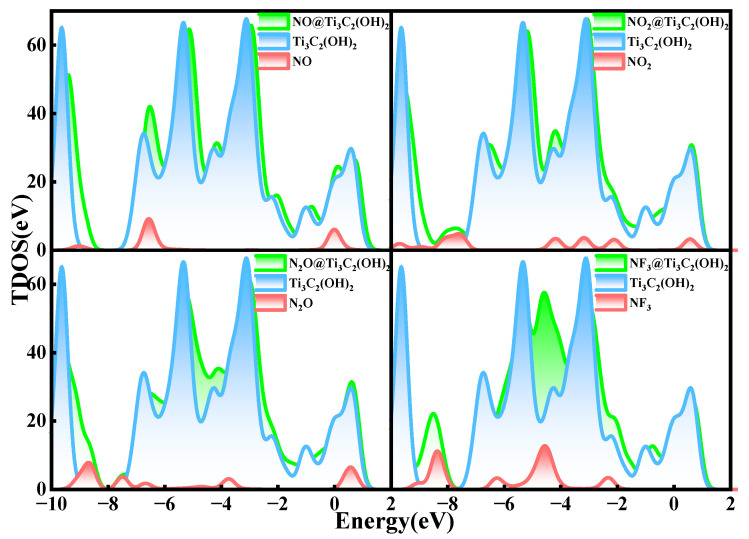
Total electron density of states of Ti_3_C_2_(OH)_2_ adsorbing NO, NO_2_, N_2_O, and NF_3_.

The TDOS of Ti_3_C_2_(OH)_2_ after gas adsorption is shown in Figure 9. Apparently, the density of states of the whole system before and after the adsorption of NO, NO_2_, N_2_O, and NF_3_ experiences a great change. It can be clearly seen that all the TDOS show a right shift, and the electronic states of the system move to higher energy levels after the adsorption of these gases, indicating that the adsorption of the gases has a relatively large effect on the adsorption system, and the densities of states all increase around the Fermi energy level, meaning that the conductivity of the material is enhanced. All these changes are consistent with the large charge transfer between the gases and Ti_3_C_2_(OH)_2_. In addition, after the adsorption of NF_3_, a new peak at −4.5 eV clearly appears, which changes the whole system the most, which also reflects the strongest interaction of NF_3_ with Ti_3_C_2_(OH)_2_. 

**Figure 10:**
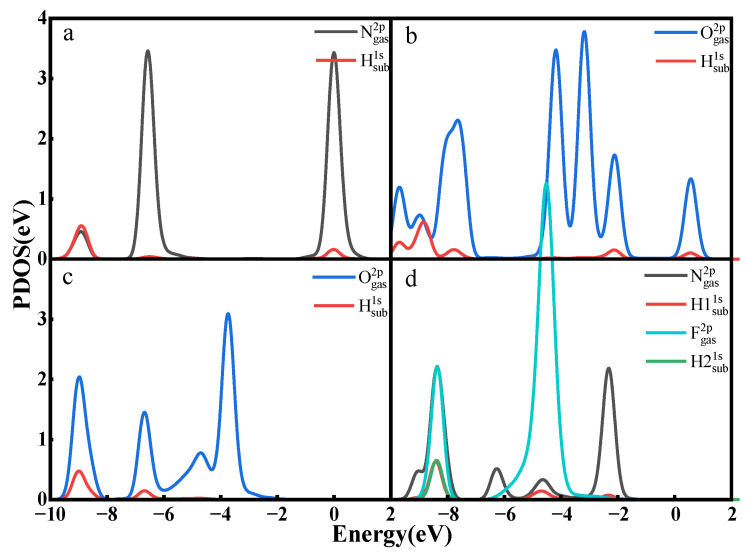
Partial electron density of states of Ti_3_C_2_(OH)_2_ adsorbing (**a**) NO, (**b**) NO_2_, (**c**) N_2_O, (**d**) NF_3_.

Figure 10 shows the PDOS for some gas atoms and surface atoms. The N 2p orbital of NO and the H 1s orbital from the surface have the same density of state peaks at 0 eV, −9 eV in Figure 10a, which indicates a resonance of the electrons in the orbital, a manifestation of a stronger interaction. The O 2p orbital of NO_2_ and the H 1s orbital at 0 eV, −2 eV, −8 eV, −9 eV have an overlap in Figure 10b, which likewise indicates a stronger interaction between them, and the O 2p orbital of N_2_O and the H 1s orbital of the surface have the same density of state peaks at −6.5 eV, −9 eV in Figure 10c, demonstrating that O–H bonds are indeed formed between O and H. The N 2p and F 2p orbitals of NF_3_ and the H1 1s and H2 1s of the surface likewise show multiple overlaps in Figure 10d, representing the formation of N–H bonds and F–H bonds.

In summary, the adsorption that occurs on the Ti_3_C_2_(OH)_2_ surface is significantly different from that of Ti_3_C_2_F_2_ and Ti_3_C_2_O_2_, which has greater adsorption energy, more charge transfer, and stronger interactions. The reason is that the hydroxyl groups on the surface are more chemically active than oxygen and fluorine atoms, and the gas molecules interact more strongly with the OH surface. When NO and NO_2_ make contact with the Ti_3_C_2_(OH)_2_ surface, N and O are attracted by OH and form O–H···N and O–H···O hydrogen bonds, respectively. When N_2_O and NF_3_ make contact with the Ti_3_C_2_(OH)_2_ surface, O atoms, N atoms, and F atoms are attracted by OH, forming O–H···O, O–H···N, and O–H···F hydrogen bonds and approaching to the surface, which eventually leads to H atom stripping from surface and forming new chemical bonds.

### 3.4. Ti_3_C_2_T_x_ Gas Sensing Performance Evaluation

An important parameter reflecting the sensing performance is the recovery time τ, which represents the time required to remove the adsorbed gas molecules from the material surface, defined as [30]

$$\tau =v_{0}^{-1}e^{\left(-\frac{E_{ads}}{kT}\right)}\;$$

$v_{0}$ indicates the attempt frequency, assuming that all gases have the same order of magnitude as that of NO_2_ (1.0 × 10^12^ s^−1^) [31]; *E_ads_* is the energy barrier for desorption, which is set equal to the adsorption energy; *k* is the Boltzmann constant (8.62 × 10^−5^ eV·K^−1^); and T is the Kelvin temperature. It can be seen from the equation that the larger the adsorption energy is for a gas molecule, the more difficult its desorption will become accordingly. The recovery time of each gas on Ti_3_C_2_T_x_ at room temperature is given in Table 3. The adsorption energies on Ti_3_C_2_F_2_ and Ti_3_C_2_O_2_ are generally small, so they reflect a fast recovery time, and the slowest is 0.37 ms for NO adsorption on Ti_3_C_2_O_2_, and the very short recovery time may not achieve effective detection in the actual sensing detection. In contrast, the adsorptions of four gases on Ti_3_C_2_(OH)_2_ have a large adsorption energy and exhibit a long recovery time. It is difficult for gas molecules to desorb from the material surface at room temperature, which is not conducive to the reuse of the sensor but has potential value as a gas adsorbent. The desired sensor performance can be achieved by surface modification. There are many ways of surface modification, such as controlling the ratio of surface F, O and OH groups, doping with other metals and their compounds, adding atomic vacancies, adding other terminal groups, etc. [32,33,34,35,36].

## 4. Conclusions

In this paper, the adsorption process of gases on the surface of these materials was studied by constructing the adsorption models of NO, NO_2_, N_2_O, and NF_3_ on Ti_3_C_2_T_x_ with three terminal groups of F, O, and OH, and the adsorption behavior of gases was analyzed according to adsorption energy, charge density, and density of states through DFT calculations:

(1) The adsorption energies of NO on Ti_3_C_2_F_2_, Ti_3_C_2_O_2_, and Ti_3_C_2_(OH)_2_ are −0.216eV, −0.507 eV, and −1.709 eV, respectively, and the charge transfers are 0.111 e, 0.284 e, and −0.607 e, respectively. After NO adsorption, the density of states of the adsorbed systems all increase near the Fermi energy level, and conductivity is enhanced.
(2) The adsorption energies of NO_2_ on Ti_3_C_2_F_2_, Ti_3_C_2_O_2_, and Ti_3_C_2_(OH)_2_ are −0.213 eV, −0.115 eV, and −3.806 eV, respectively, and the charge transfer is −0.129 e, 0.077 e, and −0.753 e, respectively, and the adsorption of NO_2_ leads to the enhancement of electrical conductivity of the materials.
(3) The adsorption energies of N_2_O on Ti_3_C_2_F_2_, Ti_3_C_2_O_2_, and Ti_3_C_2_(OH)_2_ are −0.273 eV, −0.240 eV, and −5.461 eV, respectively, with charge transfer of 0.008 e, −0.008 e, and -0.707e. After the adsorption of N_2_O, only Ti_3_C_2_(OH)_2_ exhibits an increase in electrical conductivity, and it is the chemisorption that occurs with strong interactions.
(4) The adsorption energies of NF_3_ on Ti_3_C_2_F_2_, Ti_3_C_2_O_2_, and Ti_3_C_2_(OH)_2_ are −0.323 eV, −0.386 eV, and −9.065 eV, respectively, and the charge transfers are 0.014 e, 0.036 e, and −1.428 e, respectively. NF_3_ exhibits strong chemisorption only on Ti_3_C_2_T_x_ terminated with -OH, accompanied by a large amount of charge transfer.
(5) The adsorption of all four gases on the surface of Ti_3_C_2_F_2_ and Ti_3_C_2_O_2_ exhibits short recovery times, while the adsorption on the surface of Ti_3_C_2_(OH)_2_ makes it difficult to achieve desorption at room temperature due to the stronger adsorption effect.

Combining all the analyses, Ti_3_C_2_T_x_ with three terminal groups has good sensing ability for NO and NO_2_, while Ti_3_C_2_T_x_ with -OH surface has great adsorption energy and significant charge transfer for NO, NO_2_, N_2_O, NF_3_, which makes it easy to achieve gas detection, but it is also found to have the disadvantage of long recovery time, so the performance of the material needs to be improved by further methods.

## Figures and Tables

**Table 1:** Adsorption distance and bond length change of gas molecules on Ti_3_C_2_T_x_.

Ti_3_C_2_T_x_	Gas	D(Å) ^a^	L(Å) ^b^
F	NO	2.978	−0.012
	NO_2_	2.807	0.011
	N_2_O	2.979	−0.002
	NF_3_	2.849	0
O	NO	2.769	−0.028
	NO_2_	2.806	0.005
	N_2_O	3.021	−0.001
	NF_3_	2.842	−0.002
OH	NO	1.809	0.08
	NO_2_	1.666	0.073
	N_2_O	-- ^c^	--
	NF_3_	--	--

^a^ The adsorption distance D refers to the shortest distance between the atom of gas and atom of substrate. ^b^ The bond length change L, L = L_2_−L_1_, L_1_ and L_2_ represents the chemical bond of gas before and after adsorption. A negative value indicates that the molecular bond length becomes shorter. ^c^ The -- symbol indicates that the chemical bond of the gas is broken and is not included in the measurement.

**Table 2:** Adsorption energy and charge transfer of gas molecules on Ti_3_C_2_T_x_.

Ti_3_C_2_T_x_	Gas	*E*_*ads*_(eV)	Q_t_€ ^a^
F	NO	−0.216	0.111
	NO_2_	−0.213	−0.129
	N_2_O	−0.273	0.008
	NF_3_	−0.323	0.014
O	NO	−0.507	0.284
	NO_2_	−0.115	0.077
	N_2_O	−0.240	−0.008
	NF_3_	−0.386	0.036
OH	NO	−1.709	−0.607
	NO_2_	−3.806	−0.753
	N_2_O	−5.461	−0.707
	NF_3_	−9.065	−1.428

^a^ A positive *Q*_*t*_ means that the gas molecule loses electrons, and a negative Qt means that it gains electrons.

**Table 3:** Recovery time at room temperature (25 °C).

Ti_3_C_2_T_x_	*τ*(NO)/s	*τ*(NO_2_)/s	*τ*(N_2_O)/s	*τ*(NF_3_)/s
F	4.4×10^−9^	3.9×10^−9^	4.1×10^−8^	2.8×10^−7^
O	3.7×10^−4^	8.8×10^−11^	1.1×10^−8^	3.3×10^−6^
OH	7.8×10^16^	2.2×10^52^	2.1×10^80^	1.8×10^141^

## Supplementary Materials

The following are available online at https://www.mdpi.com/article/10.3390/nano12132311/s1. Figure S1. Molecule structures of NO, NO_2_, N_2_O and NF_3_.
