# Supplementary material for: Ti3C2Tx as a Sensor for SF6/N2 Nitrogen-Containing Fault Decomposition Characteristic Products: A Theoretical Study
Source: Nanomaterials (Basel). 2022 Jul 5;12(13):2311. doi: 10.3390/nano12132311 (PMC9268492; doi:10.3390/nano12132311)
Supplement: Supplementary file 1 [file nanomaterials-12-02311-s001.zip › nanomaterials-1776569-supplementary.pdf]

nanomaterials

Supplementary Material

# $Ti_3C_2T_x$ as a Sensor for $SF_6/N_2$ Nitrogen-Containing Fault Decomposition Characteristic Products: A Theoretical Study

**Fuping Zeng [1,2,*], Hao Qiu [1], Xiaoxuan Feng [1], Xianzong Chao [1], Liangjun Dai [3], Qiang Yao [3], Ju Tang [1,2,4]**

1 School of Electrical Engineering and Automation, Wuhan University, Wuhan 430072, China; elvis_qiu@icloud.com (H.Q.); xiaoxuanf@whu.edu.cn (X.F.); 2018302070035@whu.edu.cn (X.C.); cqtangju@vip.sina.com (J.T.)
2 Hubei Key Laboratory of Power Equipment & System Security for Integrated Energy Resources, Wuhan 430072, China
3 Electric Power Research Institute, State Grid Chongqing Electric Power Company, Chongqing 401123, China; 2018302070058@whu.edu.cn (L.D.); yaoqiang212@aliyun.com (Q.Y.)
4 State Key Laboratory of Power Transmission Equipment & System Security and New Technology, Chongqing University, Chongqing 400044, China
* Correspondence: fuping.zeng@whu.edu.cn; Tel.: +86-1397-129-0926

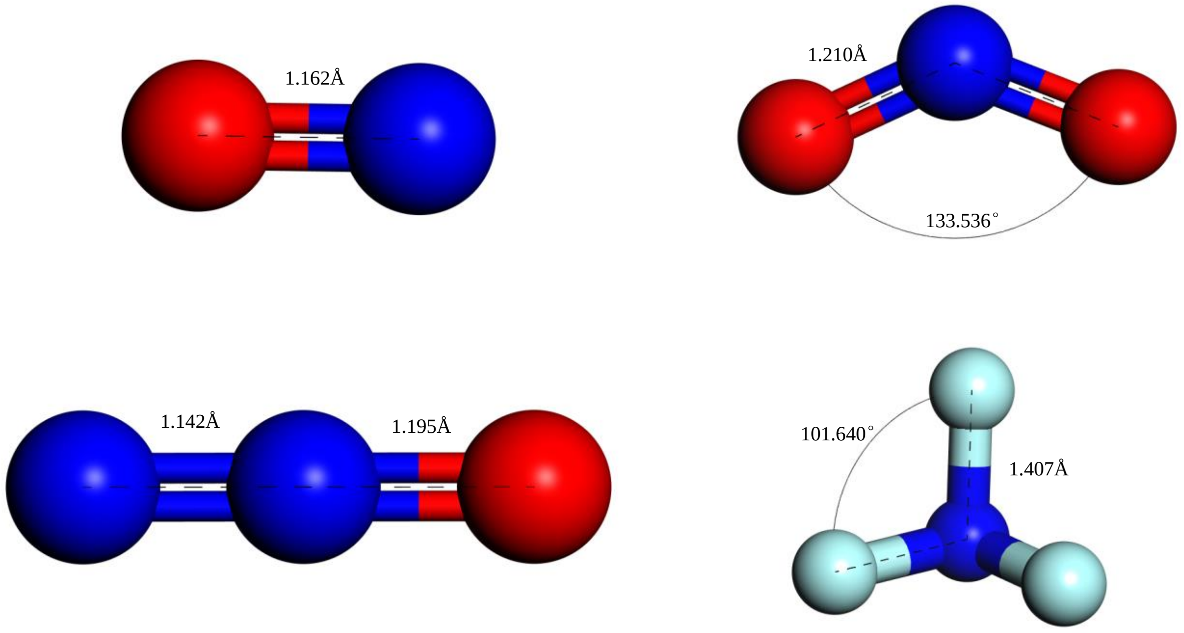


**Figure S1.** Molecule structures of NO, $NO_2$, $N_2O$ and $NF_3$.
